# Is infracolic omentectomy necessary for presumed early-stage Borderline Ovarian Tumors (BOTs)? A retrospective cohort study and meta-analysis

**DOI:** 10.1016/j.clinsp.2025.100827

**Published:** 2025-11-16

**Authors:** Rosilene Jara Reis, Lidia Rosi Medeiros, Airton Teltebom Stein, Sophie Derchain, Igor Casotti de Pádua, Leonardo Octavio Lobo Soares, Guilherme Alves Diogo da Silva, Thais Andrade Gewehr, Carlos Eduardo Mattos da Cunha Andrade, Jeferson Rodrigo Zanon, Renata Avila, Mileide M. Souza, Flavia Fazzio Barbin, Ricardo dos Reis

**Affiliations:** a Department of Gynaecologic Oncology, Santa Casa de Misericórdia de Porto Alegre, Porto Alegre, RS, Brazil; b Universidade Federal de Ciências da Saúde de Porto Alegre, Porto Alegre, RS, Brazil; c Graduate Program in Gynaecologic Oncology, Universidade Federal de Ciências da Saúde de Porto Alegre, Porto Alegre, RS, Brazil; d Family Physician at Conceição Hospital, Porto Alegre, Rio Grande do Sul, Brazil; e Department of Obstetrics and Gynaecology, Faculdade de Ciências Médicas da Universidade Estadual de Campinas (UNICAMP), Campinas, SP, Brazil; f Department of Gynaecologic Oncology, Irmandade de Santa Casa de Misericórdia de Porto Alegre, Porto Alegre, RS, Brazil; g Department of Gynaecologic Oncology, Barretos Cancer Hospital, Barretos, SP, Brazil; h Nephrology and Palliative Care at Jales Cancer Hospital, Barretos, SP, Brazil; i Department of Gynaecologic Oncology, Universidade Federal de Uberlândia, Uberlândia, MG, Brazil; j Department of Pathology-Oncology, Barretos Cancer Hospital, Barretos, SP, Brazil

**Keywords:** Ovarian tumor, Borderline ovarian tumor, Survival, Progression-free survival, Omentectomy, Recurrence

## Abstract

**Background:**

While omentectomy is included in the guidelines for the surgical management of Borderline Ovarian Tumors (BOTs), it is unclear whether removal of a normal-appearing omentum confers a therapeutic advantage.

**Methods:**

The retrospective cohort study of patients with BOTs evalua0 ted the role of routine omentectomy and was followed by a meta-analysis to enhance the robustness of the findings. Data were obtained from patients treated at three Brazilian reference centers between January 2009 and October 2023. Progression-Free Survival (PFS), risk of death, and recurrence were compared between patients who underwent omentectomy and those who did not.

**Results:**

A total of 218 patients with BOTs were assessed: omentectomy was performed in 161 (73.8 %) and not performed in 57 (26.1 %). OS at 60 months was 95.5 % in the omentectomy group and 94.6 % in the non-omentectomy group (HR = 0.97 [95 % CI 0.20‒4.68]; *p* = 0.96). PFS was 97.2 % and 89.3 %, respectively (HR = 0.42; 95 % CI 0.10‒1.76; *p* = 0.23). Twelve studies comprising 2996 women with BOT, were included in the systematic review to evaluate the outcomes with and without omentectomy. Relative Risk (RR) of recurrence was 0.94 (95 % CI 0.67‒1.31; *p* = 0.7) for the non-omentectomy group compared with the omentectomy group. No statistically significant difference was observed, with an RR of 1.98 (95 % CI 0.24‒16.43; *p* = 0.53) for risk of death and an HR of 1.02 (95 % CI 0.25‒4.15; *p* = 0.98) for PFS.

**Conclusion:**

The retrospective cohort study and meta-analysis showed a low incidence of metastatic disease in the omentum. No effect of omentectomy on OS, PFS, and recurrence in patients with BOT.

## Highlights

• The metastatic disease in the omentum was low in Borderline Ovarian Tumor (BOT).
• Omentectomy does not interfere with the risk of death and PFS in BOT.
• Omentectomy does not interfere with recurrence in BOT.

## Introduction

Borderline Ovarian Tumors (BOTs) or tumors with low malignant potential account for 10 % to 20 % of all ovarian tumors, and they are more prevalent in younger patients compared to invasive ovarian tumors.^1^ According to the International Federation of Gynecology and Obstetrics (FIGO), BOT is often diagnosed at stages I or II, with a 5-year survival rate of approximately 95 % and a 10-year survival rate of 75 % to 95 %.^1^, ^2^, ^3^ Furthermore, these tumors have been associated with late recurrence, even after several years.^2^^,^^3^ Serous borderline tumors are histologically diagnosed based on the following criteria: epithelial cell proliferation, which includes stratification of the epithelial lining of the papillae; epithelial cell multilayering; mitotic activity; and nuclear atypia, with stromal microinvasion <5 mm.^4^ In borderline endometrioid tumors, microinvasion <5 mm is difficult to detect, whereas confluent or destructive invasion greater than 5 mm is indicative of endometrioid carcinoma.^4^ Mucinous ovarian tumors remain a subject of ongoing debate in gynaecologic pathology. Borderline mucinous tumors may contain foci of intraepithelial carcinoma. Microinvasion, defined as foci smaller than 5 mm with atypia similar to that of the borderline tumor, is considered a transitional stage towards invasive carcinoma. Tumors with remarkably diffuse cytologic atypia are classified as mucinous carcinoma.^5^, ^6^, ^7^

Guidelines for the surgical management of BOTs are similar to those for ovarian cancer, encompassing hysterectomy with bilateral salpingo-oophorectomy, peritoneal washings, omentectomy, and multiple peritoneal biopsies.^8^^,^^9^ The indications for conservative surgery in stage I disease remain a matter of ongoing debate. Conservative surgery involves a thorough evaluation of the uterus, with at least part of one ovary preserved to maintain fertility.^10^

The human omentum is a folded peritoneal organ consisting of mesothelial cell layers that envelop adipose tissue. This vascularised organ regulates peritoneal fluid flow and serves as an immune cell reservoir in the peritoneal cavity. The omentum is a poorly investigated organ whose function and role in cancer are unknown. Some laboratory studies suggest the omentum may initially contribute to tumoricidal activity, which is eventually overwhelmed by the rapid proliferation of cancer cells.^11^

The omentum is a common site of metastasis in malignant ovarian cancer and can also be involved in BOTs.^12^ Therefore, the FIGO guidelines for the staging of suspected ovarian cancer recommend that all patients be subjected to infracolic omentectomy.^9^^,^^10^^,^^13^ Despite its routine use, omentectomy offers no proven advantage in terms of an increase in overall survival or a decrease in recurrence in BOTs classified as FIGO stages I–II.^14^ Furthermore, even though omentectomy is advised, it is unclear whether removal of a normal-appearing omentum confers any therapeutic advantage, particularly in light of the current availability of adjuvant chemotherapeutic agents.^15^^,^^16^ Potential complications of omentectomy include injury to the transverse colon or stomach, bleeding from ligation of the gastrocolic ligament, or possible splenic injury.^15^

Nevertheless, the indications for omentectomy in BOTs remain controversial. To critically appraise the use of omentectomy, the authors conducted a retrospective cohort study of 218 cases and systematically reviewed all relevant studies, thus offering a more objective assessment of routine omentectomy in women with BOTs.

## Materials and methods

This study was conducted and reported in accordance with the Strengthening the Reporting of Observational Studies in Epidemiology (STROBE) statement,^17^ and the systematic review was conducted following the Preferred Reporting Items for Systematic Reviews and Meta-Analyses (PRISMA) 2020 statement to ensure transparent and complete reporting.^18^ The protocol was registered with the International Prospective Register of Systematic Reviews (PROSPERO) in July 2023 (registration number CRD42023439279) at https://w.ww.crd.york.ac.uk/PROSPERO/.^19^

### Cohort study

Consecutive patients with a primary diagnosis of BOT treated at three reference centers in Brazil were assessed between January 2009 and October 2023. The study was approved and supported by the Hospital Santa Rita at Santa de Casa de Misericórdia de Porto Alegre; Barretos Cancer Hospital, state of São Paulo; and Hospital da Mulher Professor José Aristodemo Pinotti - Caism-Unicamp in Campinas, state of São Paulo. The study protocol was approved by the Research Ethics Committee of the three hospitals (registered with the Brazil platform under CAAE number: 95,374,317.0.1001.5335). The waiver of informed consente is justified based on the following grounds: i) The study is a retrospective observational, analytical, or descriptive investigation, relying solely on information obtained from medical records, institutional information systems, and/or other available clinical data sources within the institution, without the use of biological material; ii) All data were analyzed anonymously, without any nominal identification of research participants; iii) The study results were presented in aggregate form, precluding the individual identification of participants.

Patients included in this study met the following criteria: 1) Admission to the three referenced centers from January 2009 to October 2023 for primary surgical management of BOTs; and 2) Histopathological confirmation of BOT diagnosis. The exclusion criteria were as follows: 1) Diagnosis of other ovarian carcinomas; 2) Preoperative cancer treatment, including chemotherapy and radiotherapy; 3) Cancer in other organs; and 4) Missing data.

Medical records were reviewed, including patient charts, operative reports, pathology findings, and follow-up data. The dataset included: 1) Demographic and clinical variables, such as patient age and preoperative CA 125; 2) Surgical data, including the type of procedure performed (cystectomy, unilateral or bilateral salpingo-oophorectomy, hysterectomy, omentectomy, peritoneal biopsies, lymphadenectomy, and appendectomy); 3) Pathological parameters, such as tumor histology (serous, mucinous, serous-mucinous, or endometrioid), presence of invasive or non-invasive implants, and cytology findings; and 4) Outcome measures, including postoperative complications, recurrence, Overall Survival (OS), and Progression-Free Survival (PFS). Staging was adapted from the 2021 update of the FIGO classification system for ovarian cancer.^3^

### Systematic review and meta-analysis

The following databases were searched for relevant published or unpublished trials prior to September 2024: Cochrane Central Register of Controlled Trials (CENTRAL), Cochrane Library (latest issue), MEDLINE via PubMed, Embase, LILACS, CINAHL, Scopus, and Web of Science. The grey literature was also searched. There were no language restrictions, and translations were obtained whenever necessary. Two authors (LRM and RJR) independently assessed the eligibility of the retrieved full‐text reports/publications. Any disagreements were resolved by discussion or, if necessary, by consulting a Third Author (ATS).

This systematic review included cohort studies that aimed to evaluate the efficacy and safety of non-omentectomy vs. omentectomy in the management of BOTs. Comments, case-control studies, case reports, letters to the editor, and book chapters were excluded from the review. Participants were aged ≥ 18-years and had been diagnosed with early-stage BOTs according to the FIGO classification system. The intervention group did not undergo infracolic omentectomy for early-stage BOTs, as classified by FIGO stage I and II. The comparator group included patients subjected to infracolic omentectomy for BOTs at early FIGO stages. Overall survival and PFS were used as primary outcome measures. The recurrence index was assessed as a secondary outcome measure, in line with the methodology described in the Cochrane Handbook.^20^ The search strategy was based on Boolean operators “OR” and “AND”, also including Medical Subject Headings (MeSH) terms and free-text terms ((ovarian neoplasms) OR (ovarian tumour) OR (ovarian cancer)) ((omentectomy) OR (omentum) OR (epiploon) AND (survival) AND (recurrence) (MEDLINE strategies in Supplementary Material 1. In addition, a reference list of all available original articles and relevant reviews was searched manually.

### Statistical analysis

Data were analysed using Statistical Package for Social Sciences (SPSS) version 28 was used for analysis.^21^ Sample size was calculated using WinPEPI (Programs for Epidemiologists for Windows) version 11.57 and based on the study performed by Trillsch et al.^22^ and Guo et al.^23^ Considering a 5 % significance level, an 80 % statistical power, a 12.3 % rate of neoplastic omental involvement, and a minimum Hazard Ratio (HR) of 2.3 for a five-year survival period, a sample of at least 198 patients was required.

Descriptive analyses were conducted for all included patients. Patient characteristics, tumor characteristics, and operative findings were compared using Student’s *t*-test or analysis of variance (ANOVA) followed by Tukey’s test, depending on the number of categories of the categorical variable. In case of asymmetry, the Mann-Whitney or Kruskal-Wallis tests, followed by Dunn’s test, were used for continuous variables. Fisher’s exact test or chi-square tests were used for categorical variables. The adjusted residuals test was used as a complement to the analyses. The Kaplan-Meier survival curve and log-rank tests were used to compare OS and PFS in relation to omentectomy. PFS was defined as the time elapsed from the date of primary surgery to the detection of recurrence or of the most recent observation. OS was defined as the time elapsed from the date of primary surgery to death or of the most recent observation. A multivariate Cox regression model was applied to control for confounding factors. The inclusion criterion for the multivariate analysis was a p-value < 0.20 in the bivariate analysis. Cox proportional hazards regression with multiple variables was performed to estimate HR and 95 % Confidence Intervals (95 % CI) for association with omentectomy. All statistical tests were two-sided, with a significance level set at *p* < 0.05.

The treatment effects in this meta-analysis were measured using HR with a 95 % CI for time-to-event data, and Relative Risk (RR) with a 95 % CI for dichotomous outcomes was analyzed in Review Manager (RevMan Web 2020).^24^ The risk of bias of included studies was evaluated using the Risk of Bias in Non-Randomized Studies of Interventions (ROBINS-I) tool for non-randomized studies.^25^ In cases of variability in participants, populations, or interventions across the included studies, or when statistical heterogeneity was significant (*I*^2^ >50 %), the inverse variance method was utilized to implement a random‐effects model for the meta‐analysis.^26^ A sensitivity analysis was conducted by sequentially omitting one study each time and recalculating the pooled RR to assess the stability and reliability of the overall pooled results.^27^ A separate summary of the findings table was created for all the outcomes. The GRADE approach was used to assess the certainty of evidence related to the primary and secondary outcomes, as specified in the outcome measures.^28^ A two-sided p-value of < 0.05 was considered statistically significant.

## Results

### Cohort study ‒ patient characteristics

A total of 218 patients met the inclusion criteria. The mean age at diagnosis was 47.5 years (SD ±19.3) and did not include pediatric cases; Body Mass Index (BMI) was 27.8 kg/m^2^ (SD ±5.4). Serous borderline tumors accounted for 49.5 %, mucinous borderline tumors for approximately 43.1 %, serous-mucinous tumors for 6.4 %, and endometrioid tumors for 0.9 % (Table 1). Median follow-up was 44.3-months (IQR 14.6‒89). The demographic and clinicopathologic characteristics are presented in Table 1. Surgery was performed on 180 patients in stage I (82.6 %), 12 patients in stage II (5.5 %), and 26 patients in stage III (11.9 %).

**Table 1:** Clinical characteristics of patients with or without omentectomy in BOTs.

**Variable**	**Total Sample** **(*n* = 218)**	**With Omentectomy** **(*n* = 161)**	**Without Omentectomy (*n* = 57)**	**p**
**Age in years (±SD)**	47.5 ± 19.3	45.9 ± 15.7	51.9 ± 26.7	0.041^a^
**BMI (kg/m^2^) (±SD)**	27.8 ± 5.4	27.7 ± 5.4	28.7 ± 5.7	0.580^a^
**Histology – n (%)**				<0.001^b^
Serous	108 (49.5)	87 (54.0)*	21 (36.8)	
Mucinous	94 (43.1)	69 (42.9)	25 (43.9)	
Endometrioid	2 (0.9)	0 (0.0)	2 (3.5)*	
Serous-mucinous	14 (6.4)	5 (3.1)	9 (15.8)*	
**FIGO staging – n (%)**				0.001^b^
I	180 (82.6)	124 (77.0)	56 (98.2)*	
II	12 (5.5)	12 (7.5)*	0 (0.0)	
III	26 (11.9)	25 (15.5)*	1 (1.8)	
**Median CA-125 U/mL (P_25_–P_75_)**	43 (18 – 165)	48 (18.6 – 159)	37.7 (15.4 – 279)	0.537^c^
**Surgical procedure – n (%)**				
Cystectomy	7 (3.2)	3 (1.9)	4 (7.0)	0.078^d^
Unilateral salpingo-oophorectomy	60 (27.5)	41 (25.5)	19 (33.3)	0.332^b^
Bilateral salpingo-oophorectomy	151 (69.2)	114 (64.6)	37 (64.9)	1000^b^
Hysterectomy	151(69.2)	125 (77.6)	26 (45.6)	0.011^b^
Peritoneal biopsies	70 (32.1)	69 (42.9)	1 (1.8)	<0.001^b^
Lymphadenectomy	49 (22.5)	11 (47.8)	38 (19.5)	0.005^b^
Appendectomy	18 (8.3)	18 (11.2)	0 (0.0)	0.004^d^
**Surgical methods – n (%)**				0.877^b^
Laparotomy	157 (72.0)	115 (71.4)	42 (73.7)	
Laparoscopy	61 (28.0)	46 (28.6)	15 (26.3)	
**Complications – n (%)**	32 (14.7)	24 (14.9)	8 (14.0)	1000^b^
Intraoperative	14 (6.4)	7 (4.3)	7 (12.3)	0.055^d^
Postoperative	22 (10.1)	18 (11.2)	4 (7.0)	0.522^b^
**Peritoneal biopsies – n (%)**	138 (63.3)	124 (77.0)	14 (24.6)	<0.001^b^
**Peritoneal implants – n (%)**				0.002^b^
Yes	14 (6.4)	14 (8.7)*	0 (0.0)	
No	195 (89.4)	144 (89.4)	51 (89.5)	
Ignored	9 (4.1)	3 (1.9)	6 (10.5)*	
**Adjuvant treatment – n (%)**	28 (12.8)	27 (16.8)	1 (1.8)	0.007^b^
Chemotherapy	25 (11.5)	24 (14.9)	1 (1.8)	0.015^d^
**Recurrence – n (%)**	10 (4.6)	7 (4.3)	3 (5.3)	0.724^b^
**Follow-up (months) – median (P_25_–P_75_)**	44.3 (14.6 – 89)	51.3 (16.9 – 95.4)	30.9 (9.3 – 63.6)	0.010^c^
**Patient status in last appointment – n (%)**				0.159^b^
Alive and without disease	168 (77.1)	128 (79.5)	40 (70.2)	
Alive and with disease	4 (1.8)	4 (2.5)	0 (0.0)	
Death from disease	7 (3.2)	5 (3.1)	2 (3.5)	
Death from other causes	2 (0.9)	2 (1.2)	0 (0.0)	
Loss to follow-up	37 (17)	22 (13.7)	15 (26.3)	
**Surgical time (±SD)**	183.4 ± 83.7	194.3 ± 85.0	128.3 ± 48.8	<0.001^a^
**Abdominal cytology – n (%)**				0.086^b^
Positive	13 (6.0)	13 (8.1)	0 (0.0)	
Negative	165 (75.7)	119 (73.9)	46 (80.7)	
Not performed	40 (18.3)	29 (18.0)	11 (19.3)	
**Omental involvement – n (%)**				0.009^b^
Yes	21 (9.6)	21 (13)	0 (0.0)	
No	197 (90.4)	140 (87.0)	57 (100)	
**Type of omental involvement – n (%)**				0.016^b^
Invasive	5 (2.3)	5 (3.1)	0 (0.0)	
Noninvasive	16 (7.3)	16 (9.9)*	0 (0.0)	
Not affected	197 (90.4)	140 (87)	57 (100)*	
**Lymphadenectomy biopsy – n (%)**				<0.001^b^
Positive	11 (5.0)	11 (6.8)*	0 (0.0)	
Negative	38 (17.4)	38 (23.6)*	0 (0.0)	
Not performed	169 (77.5)	112 (69.6)	57 (100)*	

⁎ Statistically significant association by the residual test adjusted to 5 % significance.

a Student’s *t*-test.

b Pearson’s Chi-Square test.

c Mann-Whitney test.

d Fisher’s exact test.

### Cohort study ‒ treatment outcomes

The initial surgical approach was laparotomy in 72 % of cases or laparoscopy in 28 %. Bilateral salpingo-oophorectomy, unilateral salpingo-oophorectomy, and cystectomy were performed in 64.7 %, 27.5 %, and 3.2 % of cases, respectively. Overall, 151 patients (69.2 %) underwent total abdominal hysterectomy with bilateral salpingo-oophorectomy, whereas 67 (30.7 %) patients underwent fertility-sparing surgery. Omentectomy was performed on 161 patients (73.8 %), and omental involvement was observed in 21 patients (9.6 %), and the disease was invasive in five cases (2.3 %). In the non-omentectomy group, 98.2 % of patients were classified as FIGO stage I, whereas only 1.8 % were diagnosed with stage III. Conversely, in the omentectomy group, 124 (77 %) patients were classified as FIGO stage I, 12 (7.5 %) as stage II, and 25 (15.5 %) as stage III. Appendectomy was performed on 18 patients (8.3 %), with appendiceal involvement in only three patients (1.3 %) with mucinous tumors. Lymphadenectomy was performed on 49 patients (22.5 %), and 11 (5 %) had positive lymph nodes. Abdominal cytology was positive in 13 cases (6 %). In the non-omentectomy group, the risk of recurrence was 5.3 % (*n* = 3) compared with 4.3 % (*n* = 7) in the omentectomy group (*p* = 0.72) (Table 1).

**Fig. 1:**
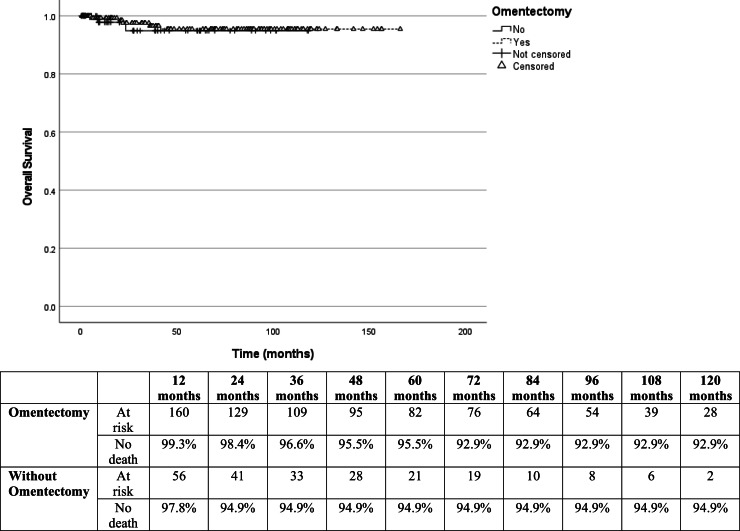
Kaplan-Meier survival curves for comparison of overall survival after omentectomy in patients with borderline ovarian tumors (Log-rank test HR = 0.97; 95 % CI 0.20‒4.7; *p* = 0.96).

**Fig. 2:**
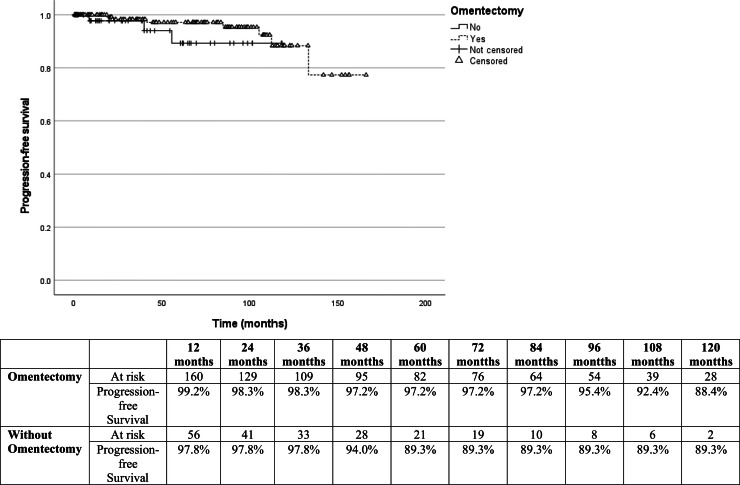
Kaplan-Meier survival curves for comparison of progression-free survival in patients with and without omentectomy (Log-rank test HR = 0.42; 95 % CI 0.10‒1.76; *p* = 0.22).

OS at 60-months was 95.5 % in patients who underwent omentectomy and 94.6 % in those who did not (Log-rank test HR = 0.97; 95 % CI 0.20‒4.68; *p* = 0.966) (Fig. 1). PFS was 97.2 % in patients who had omentectomy compared to 89.3 % in those who did not (Log-rank test HR = 0.42; 95 % CI 0.10‒1.76; *p* = 0.233) (Fig. 2). Nonetheless, owing to a wide 95 % CI and *p* > 0.05, additional information is needed to thoroughly evaluate this outcome. The log-rank test revealed no statistically significant difference between the OS and PFS analyses. Univariable Cox regression analysis was performed to evaluate independent factors associated with OS and PFS in BOT, and only FIGO stage III had an important prognostic impact (Log-rank test HR = 5.23; 95 % CI 1.30‒20.9; *p* = 0.020) (Table 2). After adjusting for confounding variables using the multivariate Cox regression model, FIGO stage III was identified as the sole independent risk factor associated with death (Log-rank test HR = 5.99; 95 % CI: 1.19‒30.2; *p* = 0.030) and recurrence (Log-rank test HR = 4.06; 95 % CI: 1.11–14.9; *p* = 0.035) (Table 3).

**Table 2:** Univariate Cox regression model to assess factors independently associated with death and recurrence in patients with BOTs.

**Variable**	**Overall survival**			**Progression-free survival**		
	**N Death (%)**	**HR (95% CI)**	**p**	**N Recurrence (%)**	**HR (95% CI)**	**p**
**Omentectomy**						
Yes	7 (4.3)	0.97 (0.20‒4.68)	0.966	7 (4.3)	0.42 (0.10‒1.76)	0.236
No	2 (3.5)	1.00		3 (5.3)	1.00	
**FIGO staging**						
I	4 (2.2)	1.00	‒	5 (2.8)	1.00	‒
II	1 (8.3)	2.79 (0.31‒25.0)	0.360	0 (0.0)	a	a
III	4 (15.4)	5.23 (1.30‒20.9)	0.020	5 (19.2)	3.54 (0.98 – 12.8)	0.053
**CA-125 U/mL**	‒	1.00 (0.99‒1.00)	0.840	‒	1.00 (0.99‒1.00)	0.354
**Hysterectomy**						
Yes	7 (5.3)	1.94 (0.40‒9.36)	0.408	4 (3.0)	0.39 (0.11‒1.39)	0.148
No	2 (2.3)	1.00		6 (7.0)	1.00	
**Complications**						
Yes	2 (6.3)	1.75 (0.36‒8.42)	0.485	1 (3.1)	0.55 (0.07‒4.36)	0.569
No	7 (3.8)	1.00		9 (4.8)	1.00	
**Intraoperative complications**						
Yes	1 (7.1)	1.90 (0.24‒15.2)	0.546	1 (7.1)	1.32 (0.17‒10.5)	0.795
No	8 (3.9)	1.00		9 (4.4)	1.00	
**Postoperative complications**						
Yes	1 (4.5)	1.27 (0.16‒10.2)	0.821	0 (0.0)	0.04 (0.00‒303)	0.483
No	8 (4.1)	1.00		10 (5.1)	1.00	
**Abdominal cytology**						
Positive	1 (7.7)	1.62 (0.20‒13.5)	0.654	0 (0,0)	a	a
Negative	6 (3.6)	1.00		7 (4.2)	1.00	
Not affected	2 (5.0)	1.62 (0.33‒8.01)	0.557	3 (7.5)	1.86 (0.48‒7.24)	0.371
**Lymphadenectomy biopsy**						
Positive	1 (9.1)	1.41 (0.17‒11.8)	0.753	1 (9.1)	0.62 (0.08‒5.13)	0.656
Negative	2 (5.3)	1.07 (0.22‒5.31)	0.936	1 (2.6)	0.28 (0.03‒2.28)	0.233
Not performed	6 (3.6)	1.00		8 (4.7)	1.00	
**Age at diagnosis** (Years)	‒	1.01 (0.99‒1.03)	0.427	‒	1.01 (0.99‒1.03)	0.612
**BMI (kg/m^2^**)	‒	1.05 (0.89‒1.23)	0.570	‒	1.05 (0.89‒1.23)	0.558
**Histology**						
Serous	3 (2.8)	1.00	‒	5 (4.6)	1.00	‒
Mucinous	5 (5.3)	2.29 (0.55‒9.59)	0.257	5 (5.3)	1.68 (0.47‒5.93)	0.423
Endometrioid	0 (0.0)	a	a	0 (0.0)	a	a
Serous-mucinous	1 (7.1)	2.80 (0.29‒27.0)	0.372	0 (0.0)	a	a
**Surgical methods**						
Laparotomy	5 (3.2)	0.50 (0.13‒1.85)	0.298	7 (4.5)	0.83 (0.21‒3.21)	0.781
Laparoscopy	4 (6.6)	1.00		3 (4.2)	1.00	
**Type of omental involvement**						
Invasive	1 (20.0)	4.95 (0.61‒40.4)	0.136	1 (20.0)	2.54 (0.30‒21.7)	0.396
Noninvasive	1 (6.3)	1.34 (0.16‒10.9)	0.785	2 (12.5)	2.10 (0.43‒10.2)	0.360
Not affected	7 (3.6)	1.00		7 (3.6)	1.00	

FIGO, International Federation of Gynaecology and Obstetrics; BMI, Body Mass Index; BOTs, Borderline Ovarian Tumours.

a It was not possible to estimate due to insufficient number of cases.

**Table 3:** Multivariate Cox regression models for evaluation of independent factors associated with death and recurrence in patients with BOTs.

**Variable**	**Overall survival**		**Progression-free survival**	
	**HR (95% CI)**	**p**	**HR (95% CI)**	**p**
**Type of omental involvement**				
Invasive	1.27 (0.12‒13.4)	0.845	‒	‒
Noninvasive	0.50 (0.05‒4.79)	0.544	‒	‒
Not affected	1.00		‒	‒
**Hysterectomy**	‒	‒	0.33 (0.09‒1.18)	0.088
**FIGO staging**				
I	1.00	‒	1.00	‒
II	2.89 (0.32‒26.0)	0.343	a	a
III	5.99 (1.19‒30.2)	0.030	4.06 (1.11–14.9)	0.035

FIGO, International Federation of Gynaecology and Obstetrics; BOTs, Borderline Ovarian Tumours.

a It was not possible to estimate due to insufficient number of cases.

### Systematic review and meta-analysis

#### Included trials

**Fig. 3:**
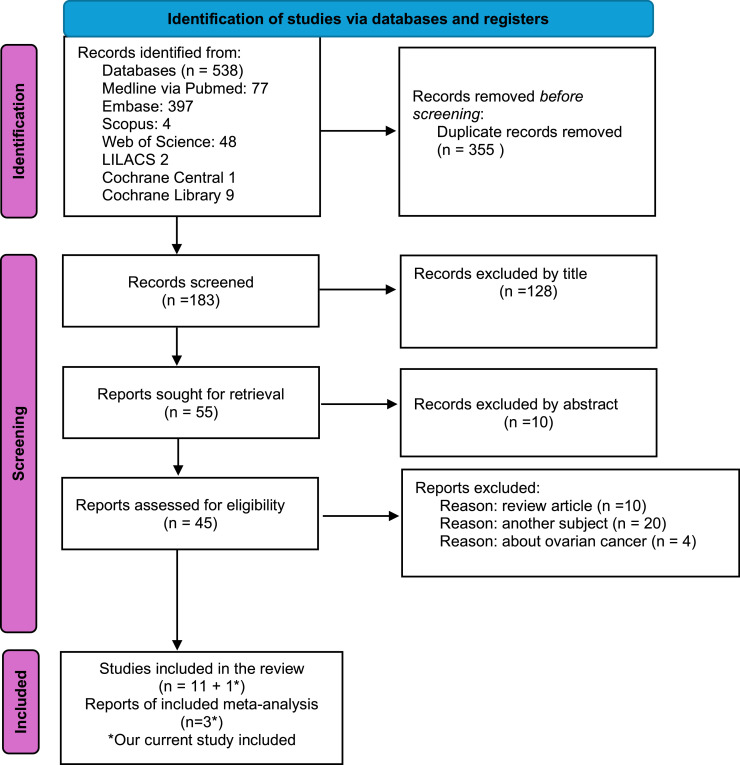
PRISMA 2020 flow diagram of the study process. From: Page MJ, McKenzie JE, Bossuyt PM, Boutron I, Hoffmann TC, Mulrow CD, et al. The PRISMA 2020 statement: an updated guideline for reporting systematic reviews. BMJ 2021;372:n71. doi: 10.1136/bmj.n71 (24).

Fig. 3 shows the screening and selection processes, as outlined in the PRISMA flow diagram.^18^ A total of 538 records were retrieved from the database search. After screening the titles and abstracts, 538 articles were initially identified, and 77 non-relevant studies were excluded. In addition, 355 duplicate studies were excluded. The remaining 106 records were carefully reviewed after title and abstract screening. Forty-five studies were retrieved for full-text screening based on the established exclusion and inclusion criteria. A total of 12 studies, including the authors’ own, evaluated surgical management with and without omentectomy in cases of BOT (Fig. 3) .^8^^,^^13^^,^^22^, ^23^^,^^29^, ^30^, ^31^, ^32^, ^33^, ^34^, ^35^ All of the included studies were retrospective cohort studies, and their characteristics are summarised in Table 4.

**Table 4:** Characteristics of included studies.

**Author**	**Country**	**Type of study**	**Data collection**	**Sample size**	**Duration Follow-up**	**Statistical method**	**Outcomes**	**Comparision Group**	**Overall bias (ROBINS-I)**
Trillsch^22^ 2015	Germany	Retrospetive cohort (24 centres)	1998‒2008	559	60 months	Cox Regression	Recurrence	Omentectomy vs. Non omentectomy	Serious risk
							Risk of death		
						Kaplan-Meier	PFS		
Guo^23^ 2023	China	Retrospective cohort	2003‒2018	901	62 months	Cox regression	Recurrence	Omentectomy vs. Non omentectomy	Serious risk
						Kaplan-Meier			
Current study 2025	Brazil	Retrospective cohort (3 centres)	2009‒2023	218	44 months	Cox regression	Recurrence	Omentectomy vs. Non omentectomy	Serious risk
						u	OS		
						Chi-Square	PFS		
Bendifallah^8^ 2015	France	Retrospective cohort (2 centres)	1980‒2008	428	94 months	Chi-Square	Recurrence	No comparision	Critical risk
						Mann-Whitney	OS		
						Fisher’s test	PFS		
Gungorduk^13^ 2017	Turkey	Retrospective cohort (14 centres)	1998‒2015	364	53 months	Cox regression	Recurrence	Non comparision	Critical risk
							OS		
						Kaplan-Meier	PFS		
Loizzi^29^ 2015	Italy	Retrospective cohort	1991‒2011	55	60 months	Kaplan-Meier	Recurrence	No comparision	Critical risk
						Chi-square	OS		
							PFS		
Ye^30^ 2022	China	Retrospective cohort	2014‒2017	74	48 months	Cox regression	Recurrence	No comparision	Critical risk
						Kaplan-Meier	OS		
						Chi-Square	PFS		
Kipp,^31^ 2023	Switzerland	Retrospective cohort	2011‒2018	42	52 months	Chi-Square	Recurrence	No comparision	Critical risk
						Student’s test	Death		
De Decker^32^ 2017	Netherlands	Retrospective cohort	1990‒2015	74	46 months	Not describe	Recurrence	No comparision	Critical risk
Trillsch^33^ 2013	Germany	Retrospective cohort	1993‒2008	105	Not reported	Chi-Square	Type surgery	No comparision	Critical risk
						Fisher’s test	Complications		
Cammatte^34^ 2004	France	Retrospective cohort	1965‒1998	101	75 months	Chi-Square	Recurrence	No comparision	Serious risk
						Fisher’ test			
Kristensen^35^ 2014	Denmark	Retrospective cohort	2007‒2011	75	28 months	Chi-Square	Recurrence	No comparision	Critical risk
						Fisher’s test Sensitivity	Surgion ability to identify tumor spread		
						Specificity			

OS, Overall Survival; PFS, Progression-Free Survival.

In total, 2996 cases of BOT were assessed. Omentectomy was performed in 1766 (58.9 %) cases and not performed in 1230 (41.1 %) cases. Eight studies were carried out at a single centre^23^^,^^29^, ^30^, ^31^, ^32^, ^33^, ^34^, ^35^; one trial at two centres^8^; one trial at 14 centres^13^; and one trial at 24 centres^22^; The current study was conducted at three centres. Two included trials were conducted in Germany,^22^^,^^33^ China^23^^,^^30^ and France.^8^^,^^34^ The other six studies were from Italy,^29^ Switzerland,^31^ the Netherlands,^32^ Turkey,^13^ Denmark,^35^ and Brazil (current study) (Table 4).

Nine studies included in this systematic review did not compare the outcomes between the non-omentectomy and omentectomy groups, but merely reported whether omentectomy was performed in cases of BOT.^8^^,^^13^^,^^29^, ^30^, ^31^, ^32^, ^33^, ^34^, ^35^ A total of 1318 cases were analyzed, including 570 patients who underwent omentectomy. Among these, 49 (8.5 %) cases showed omental involvement.^8^^,^^13^^,^^29^, ^30^, ^31^, ^32^, ^33^, ^34^, ^35^ The median age was 45.7 years (range 13‒90) in eight studies.^8^^,^^13^^,^^29^, ^30^, ^31^^,^^33^, ^34^, ^35^ The histological subtypes of 880 patients were evaluated across seven included studies.^8^^,^^29^, ^30^, ^31^^,^^33^, ^34^, ^35^ Serous tumors were identified in 472 (53.6 %) women, mucinous tumors in 378 (42.9 %), serous-mucinous tumors in 20 (2.2 %), endometrioid tumors in 6 (0.68 %), and Brenner tumor in 1 (0.11 %).^8^^,^^29^, ^30^, ^31^^,^^33^, ^34^, ^35^ The median follow-up time was 60.5 months (range 1‒207.3) in six studies.^8^^,^^29^^,^^31^, ^32^^,^^34^, ^35^ In five studies with a total of 983 patients, 736 (74.8 %), 55 (5.6 %), and 192 (19.5 %) patients were classified as FIGO stages I, II, and III, respectively.^8^^,^^13^^,^^30^, ^31^^,^^35^ Recurrence occurred in 161 cases (13.2 %) in eight studies with a total of 1213 cases.^8^^,^^13^^,^^29^, ^30^, ^31^, ^32^^,^^34^, ^35^ The study with higher recurrence rates (101 patients) had more patients classified as FIGO stage III (137 cases) .^5^ Cox regression analysis showed that only TNM stages were significantly associated with PFS.^30^

#### Meta-analysis

Three studies, including our own, comprising a total of 1678 patients, examined the association of non-omentectomy and omentectomy with risk of death, PFS, and recurrence in patients with BOT.^22^^,^^23^ The authors performed a meta-analysis, including our current study (Table 4). The median age in these three studies was 44.9 years (range 11‒92) .^22^^,^^23^ Data from three studies involving 1702 patients who underwent surgical staging according to the FIGO classification revealed that 1362 (81.2 %) patients were stage I, 131 (7.8 %) were stage II, and 209 (12.5 %) were stage III.^22^^,^^23^ Omental involvement occurred in 222 cases (11.2 %).^22^^,^^23^ Recurrence occurred in 188 cases (13.2 %),^22^^,^^23^ and the study with higher recurrence (125 patients) had more patients in FIGO stage III (81 cases) .^22^

#### Primary outcomes in the meta-analysis

**Fig. 4:**
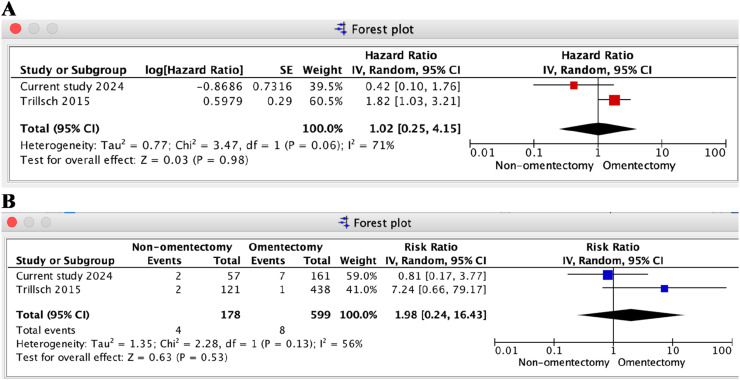
Forest plots of the meta-analysis for progression-free survival and overall survival outcomes between non-omentectomy and omentectomy in borderline ovarian tumor. (A) Forest plot of progression-free survival (hazard ratio); (B) Forest plot of risk of death(relative risk).

Two studies, including the authors’ own, reported data on PFS and risk of death in BOT, comparing the non-omentectomy and omentectomy groups.^22^ The results of the meta-analysis revealed a non-statistically significant difference in PFS (HR = 1.02, 95 % CI 0.25–4.15, *I*^2^ = 71 %, *p* = 0.98) between the two groups (Fig. 4A). Overall survival could not be calculated because one of the studies included in the meta-analysis did not report Hazard Ratios (HR). Multiple attempts to contact the authors via email were unsuccessful. Consequently, the risk of death (RR) at 60-months was calculated instead. The results did not show a statistically significant difference between the two groups (RR = 1.98; 95 % CI: 0.24–16.43; *I*^2^ = 56 %; *p* = 0.53) (Fig. 4B). However, the wide 95 % Confidence Interval underscores the need for additional data, and the p-value for this outcome was greater than 0.05.

#### Secondary outcomes in the meta-analysis

**Fig. 5:**
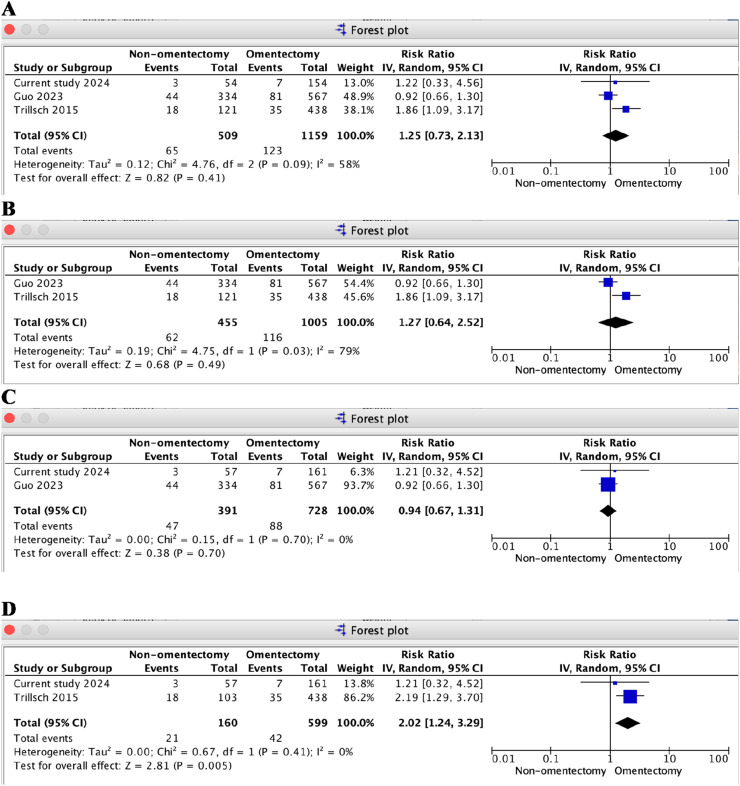
Forest plot in multiple sensitivity analysis of meta-analysis for recurrence outcome between non-omentectomy and omentectomy in borderline ovarian tumor. (A) Three studies; (B) Exclusion of the current study; (C) Exclusion of the study by Trillsch et al.^20^; (D) Exclusion of the study by Guo et al. ^21^.

In the random effects model, the estimated rate of recurrence was 14.5 % in the non-omentectomy group and 11.8 % in the omentectomy group. An RR of 1.25 was found in three studies, including the authors’ own, for the non-omentectomy and omentectomy groups (95 % CI 0.73‒2.13, *I*^2^ = 58 %, *p* = 0.41) (Fig. 5A) .^22^^,^^23^

#### Subgroup and sensitivity analysis

Subgroup analysis could not be performed, as the included studies did not provide separate data. Multiple sensitivity analyses were conducted to assess whether the sequential exclusion of individual studies affected the stability of the pooled recurrence results. The exclusion of the study by Trillsch et al.^22^ changed the magnitude and significance of the pooled results, yielding an RR of 0.94 (non-omentectomy vs. omentectomy group, 95 % CI 0.67‒1.31, *I*^2^ = 0 %, *p* = 0.7) (Fig. 5C)^23^ (including the present cohort study).

### Publication bias

Tests for funnel plot asymmetry in meta-analyses should generally be conducted only when at least 10 studies are included.^36^ The assessment of publication bias for PFS, risk of death, and recurrence was not applicable due to the limited number of included studies in the meta-analysis (only three studies) .^36^

### Quality assessment

Quality assessment was carried out using the validated ROBINS-I (Risk of Bias in Non-randomized Studies of Interventions) scale (https://www.riskofbias.info/).^25^ The ROBINS-I scale provides an overall rating from low to critical risk. The authors found four studies with serious risk, including the current study,^22^^,^^23^^,^^34^ and eight studies with critical risk^8^^,^^13^^,^^29^, ^30^, ^31^, ^32^, ^33^^,^^35^ (Supplementary Fig. 6).

### Assessment of the certainty of evidence

The GRADE approach was used to evaluate certainty in the overall body of evidence.^37^ Certainty of evidence refers to how certain it is that the true effect of an intervention lies within a chosen range or on one side of a specified threshold. When not available, the authors applied GRADE ourselves and appraised the potential limitations, such as risk of bias, inconsistency, imprecision, and indirectness, based on the original studies. Low certainty was found for PFS, risk of death, and recurrence (Table 5).

**Table 5:** Summary of findings.

**Non omentectomy compared to omentectomy for borderline ovarian tumor**						
**Patient or population:** borderline ovarian tumor						
**Setting:** surgery						
**Intervention:** Non omentectomy						
**Comparison:** omentectomy						
**Outcomes**	Anticipated absolute effects*(95% CI)		**Relative effect (95% CI)**	**Nº of participants (studies)**	**Certainty of the evidence (GRADE)**	**Comments**
	**Risk with omentectomy**	**Risk with Non omentectomy**				
Progression free survival (PFS) assessed with: HR follow-up: mean 5 years	**Low**		**HR = 1.02** (0.25 to 4.15) [Progression free survival]	777 (2 non-randomised studies)	⊕⊕⃝⃝ Low^a^^,b,^^c^^,^^d^	
	0 per 1.000	**NaN per 1.000** (– to –)				
Risk of death (Risk of death) assessed with: RR follow-up: mean 5 years	**Study population**		**RR 1.98** (0.24 to 16.43)	777 (2 non-randomised studies)	⊕⊕⃝⃝ Low^e^^,^^f^^,^^g^	
	13 per 1.000	**26 per 1.000** (3 to 219)				
	**Low**					
	0 per 1.000	**0 per 1.000** (0 to 0)				
Recurrence (Recurrence) assessed with: RR follow-up: mean 5-years	**Study population**		**RR 0.94** (0.67 to 1.31)	1668 (2 non-randomised studies)	⊕⊕⃝⃝ Low^e^^,^^h^^,^^i^	
	106 per 1.000	**100 per 1.000** (71 to 139)				
	**Low**					
	0 per 1.000	**0 per 1.000** (0 to 0)				

GRADE Working Group grades of evidence.

High certainty: we are very confident that the true effect lies close to that of the estimate of the effect.

Moderate certainty: we are moderately confident in the effect estimate: the true effect is likely to be close to the estimate of the effect, but there is a possibility that it is substantially different.

Low certainty: our confidence in the effect estimate is limited: the true effect may be substantially different from the estimate of the effect.

Very low certainty: we have very little confidence in the effect estimate: the true effect is likely to be substantially different from the estimate of effect.

Explanations:.

a This is a non-randomized study.

c Large CI and high heterogeneity (71 %).

d The total sample size is not large and the number of events is small. Under these circumstances, one should consider rating down for imprecision.

e This is a non-randomized study.

f Large CI and moderate heterogeneity (56 %).

g The total sample size is not large and the number of events is small. Under these circumstances, one should consider rating down for imprecision.

h Large CI and moderate heterogeneity (58 %).

i The total sample size is not large and the number of events is small. Under these circumstances, one should consider rating down for imprecision.

⁎ **The risk in the intervention group** (and its 95 % Confidence Interval) is based on the assumed risk in the comparison group and the **relative effect** of the intervention (and its 95 % CI). CI, Confidence Interval; HR, Hazard Ratio; RR, Risk Ratio.

## Discussion

This cohort study and meta-analysis of patients with BOT revealed omental involvement in 9.6 % and 8.5 % of the patients, respectively. The extent of surgical treatment and staging for patients with BOTs remains a topic of ongoing debate. In the retrospective cohort, the authors demonstrated that surgical management without omentectomy may still provide sufficient accuracy for clinical decision-making regarding its role in Borderline Ovarian Tumors (BOTs), despite the wider confidence intervals observed for Overall Survival (OS) and Progression-Free Survival (PFS). These findings suggest that additional data are necessary to draw definitive conclusions. The current study yielded similar results to those described in the literature, including excellent prognosis with a five-year survival in patients without omentectomy.^2^^,^^22^^,^^23^ In the cohort study, multivariate Cox regression models for evaluating independent factors associated with death and recurrence in patients with BOTs showed that only the stage is significantly associated with death and recurrence. Recurrence rates were low in the retrospective cohort study. These results can be attributed to the fact that most of the 218 patients in the present sample were classified as FIGO stage I.

A meta-analysis was performed to determine the effect of non-omentectomy vs. omentectomy on patients with BOT to further validate the findings. In this meta-analysis, only three studies (including the authors’ own) compared recurrence in non-omentectomy and omentectomy groups.^22^^,^^23^ Also, two studies compared OS and PFS in the non-omentectomy and omentectomy groups^22^ (including the current study). A meta-analysis was performed to assess these outcomes. RR was used for OS because the study by Trillsch et al. did not report the HR with a 95 % CI for OS, not allowing us to calculate the HR for this outcome.^22^

The authors attempted to contact the corresponding author but did not receive a reply.^22^ Overall survival did not show statistical differences between the non-omentectomy and omentectomy groups. It should be noted that the study by Trillsch only analyzed borderline serous tumors, which have a higher risk of progressing to advanced stages, especially those of micropapillary lineage.

HR with a 95 % CI for PFS obtained in the study allowed us to calculate HR in the meta-analysis. No statistically significant difference was observed between the non-omentectomy and omentectomy groups regarding PFS in BOT.^22^ The meta-analysis showed that omentectomy does not interfere with recurrence, but this was only the result from two studies.^23^ (including the cohort study). Nevertheless, multiple sensitivity analysis was performed to evaluate whether the sequential exclusion of individual studies affected the stability of the pooled results. When the authors excluded the study by Guo et al. ,^23^ the meta-analysis showed that omentectomy protects against recurrence. This protection is likely influenced by the study of Trillsch et al. ,^22^ in which patients undergoing omentectomy (*n* = 438) were diagnosed at a higher FIGO stage (stage III, *n* = 81) compared with patients who did not undergo omentectomy (*n* = 121), and they had higher rates of peritoneal implants (26.7 %). These factors were probably associated with OS and PFS, showing a trend towards omentectomy, but with considerable heterogeneity (> 50 %) and a wide confidence interval. In the meta-analysis of recurrence rates, multiple sensitivity analyses affected the magnitude and significance of the pooled results, showing no heterogeneity (0 %), yielding relatively narrow confidence intervals.^23^

A meta-analysis using Cox models, which could have provided more precise estimates, was not feasible due to the lack of data in many studies. In nine studies, it was not possible to extract data for the meta-analysis, given that these data did not compare omentectomy and omentectomy groups in patients with BOT.^8^^,^^13^^,^^29^, ^30^, ^31^, ^32^, ^33^, ^34^, ^35^ In these nine studies with 1318 patients, 570 cases (43.2 %) underwent omentectomy, and only 49 cases (8.5 %) had positive omental involvement.^8^^,^^13^^,^^29^, ^30^, ^31^, ^32^, ^33^, ^34^, ^35^ Note that omentectomy was not performed in 748 BOT cases (56.7 %), and this selective treatment might have overlooked occult omental involvement.

The strength of the present study lies in its 14-year analysis of a rare disease managed consistently by three different centers, thereby minimizing selection bias and providing more general applicability of the findings. All consecutive patients treated during the study period at the three reference centers were included, and all cases were subjected to pathological review, resulting in a well-characterized cohort. The follow-up period for the survival analysis was long (44.3-months), which contributed to the robustness of the present data. The main strengths of the present systematic review and meta-analysis were the rigorous data collection, bias assessment, processing, and interpretation methodologies. The authors followed a preregistered protocol that included a comprehensive search strategy, specific selection criteria, and a predefined data analysis plan.^38^

This retrospective cohort study had some limitations. Firstly, the sample size of this study was relatively small, and more patients need to be included to increase power and consolidate the findings. Secondly, the retrospective design of the present study is associated with an inherent risk of selection and reporting biases. Thirdly, misclassification bias may have occurred, as the accuracy of clinical data depends on proper coding and comprehensive documentation in the medical records, which were recorded prior to the initiation of the research protocol. The systematic review included all published studies on non-omentectomy vs. omentectomy for BOT, but it had several limitations. One limitation was that pooled analysis of the data was not possible because many studies did not provide data on omentectomy in BOT. In addition, the relatively low level of quality of the included studies was evident from the observed statistical heterogeneity. A meta-analysis could only be performed in three studies, including the authors’ own.^22^^,^^23^ In the meta-analysis, the authors did not perform subgroup analysis for the long-term oncological outcomes between non-omentectomy and omentectomy in BOT because the studies did not provide separate data. On the other hand, the authors performed multiple sensitivity analyses, which altered the magnitude and significance of pooled results for recurrence when the study by Trillsch et al. was excluded.^22^^,^^23^

This retrospective cohort study and meta-analysis confirmed the low incidence of omental involvement: 9.6 % of patients had omental involvement, with 2.3 % showing invasive disease in these implants. Notably, the study does not address the potential role of omentectomy in specific high-risk subgroups, such as micropapillary serous BOTs or mucinous BOTs with intraepithelial carcinoma. Therefore, the applicability of these results in the clinical practice may be limited to low-risk, early-stage BOTs.

These findings support a more individualized approach to the management of early-stage BOTs, potentially reducing unnecessary omentectomies and their associated surgical risks. The findings highlight the importance of considering clinical factors rather than routinely performing omentectomy.

## Conclusions

Although there is a low level of certainty of evidence for several outcomes assessed in this study, the author concludes that the present study highlights the important role of the gynaecologic oncologist in identifying low-risk criteria for not performing omentectomy in patients with BOT classified as FIGO stages I and II. Patients with borderline ovarian tumors who underwent surgery with or without omentectomy showed little to no difference in risk of death (low-certainty evidence), likely little to no difference in progression-free survival (low-certainty evidence), and probably little to no difference in recurrence (low-certainty evidence). However, the present results should be interpreted with caution until further prospective, randomized controlled trials provide sufficient evidence on whether omentectomy is required for borderline ovarian tumors.

## Written informed consent

Written informed consent for publication was obtained from all patients.

## Ethics approval

The study was approved by the Institutional Review Board of ISCMPA and of the Universidade Federal de Ciências da Saúde de Porto Alegre (UFCSPA) and was registered with Plataforma Brasil (CAAE: 95,374,317.0.1001.5335).

## Authors’ contributions

RJR, LRM, ATS, RR, CEMCA, SD were responsible for the original design, drafting, and revision of important intellectual content. FFB was the pathologist who reviewed the cases of BOT. GAD, TAG, RA, and MMS were responsible for the clinical and surgical approaches. RJR is the guarantor of this article and takes full responsibility for the work and/or conduct of the study. Her involvement was critical at every stage of this work, and she also controlled the decision to publish. ICP and LOLS were responsible for the data collected at Hospital de Amor in Barretos, Hospital Santa Rita in Porto Alegre, and Hospital da Mulher Professor José Aristodemo Pinotti ‒ Caism-Unicamp ‒ in Campinas. JRZ critically revised the whole manuscript, providing important intellectual suggestions.

## Funding

None.

## Data availability statement

The datasets generated and/or analyzed during the current study are available from the corresponding author upon reasonable request.

## Declaration of competing interest

The authors declare that they have no financial or personal relationships with other people or organizations that could inappropriately influence the present work. All authors declare that they have no conflicts of interest.

## Appendix Supplementary materials

Image, application 1
