# Supplementary material for: Is infracolic omentectomy necessary for presumed early-stage Borderline Ovarian Tumors (BOTs)? A retrospective cohort study and meta-analysis
Source: Clinics (Sao Paulo). 2025 Nov 16;80:100827. doi: 10.1016/j.clinsp.2025.100827 (PMC12664605; doi:10.1016/j.clinsp.2025.100827)

**CLINICS-D-25-00868**

**Supplementary Material 1**

**Appendix 1** Medline strategies.

| 1. exp Ovarian Neoplasms/ |
| --- |
| 2. (ovar* adj5 (cancer* or tumor* or tumour* or neoplas* or carcinoma* or malignan* or adenocarcinoma*)).mp. |
| 3. 1 or 2 |
| 4. exp borderline ovarian tumour/ |
| 5. 3 and 4 |
| 6. Exp omentectomy/ |
| 7. (Omentum).mp |
| 8. (epiploon).mp |
| 9. 6 or 7 or 8 |
| 10. 5 and 9 |
| 11. randomized controlled trial.pt. |
| 12.. controlled clinical trial.pt. |
| 13. randomized.ab. |
| 14.. placebo.ab. |
| 15. clinical trials as topic.sh. |
| 16. randomly.ab. |
| 17. trial.ti. |
| 18. Non‐Randomized Controlled Trials as Topic/ |
| 19. (quasi adj2 stud*).mp. |
| 21. exp Cohort Studies/ |
| 22. (cohort* adj2 stud*).mp. |
| 23. 11 or 12 or 13 or 14 or 15 or 16 or 17 or 18 or 19 or 20 or 21 or 22 |
| 24. 10 and 23 |
| 25. exp animals/ not humans.sh. |
| 26. 24 not 25 |
| 27. Exp survival/ |
| 28. Exp recurrence/ |
| 29. Exp “quality of life”/ |
| 30. Exp Adverse events/ |
| 31. 27 or 28 or 29 or 30 |
| 32. 26 and 31 |
| Key: |
| mp = title, abstract, original title, name of substance word, subject heading word, protocol supplementary concept, rare disease supplementary concept, unique identifier |
| pt = publication type |
| ab = abstract |
| sh = subject heading |
| ti = title |

**Supplementary** **Figure 6** Risk of bias assessment for non-randomized studies (ROBINS-I). ROBINS-I, Risk of Bias in Non-randomized Studies of Interventions.


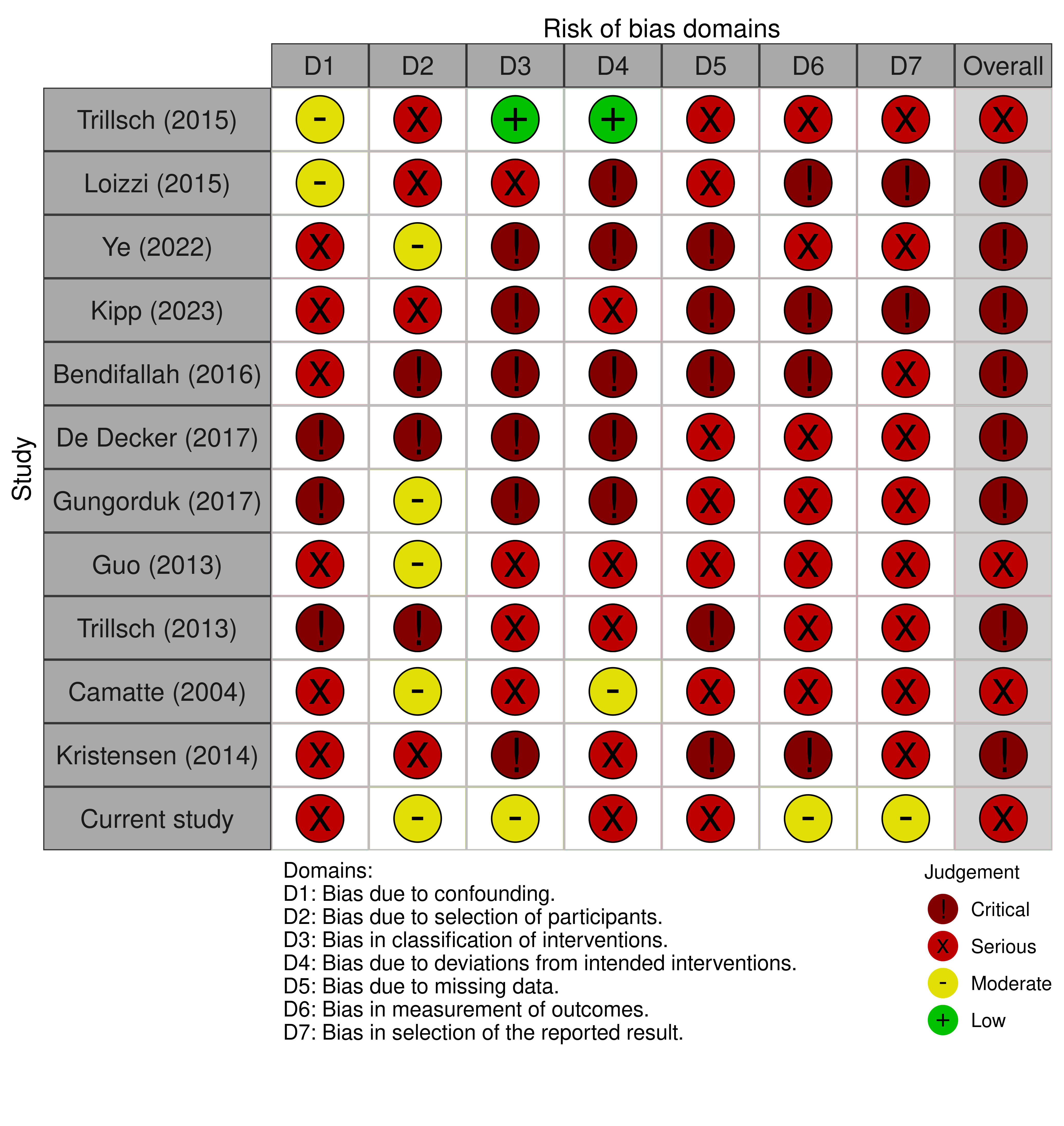

Supplement: Supplementary file 1 [file mmc1.docx]
